# Concurrent Presentation of Emphysematous Pyelonephritis, Emphysematous Osteomyelitis, and Psoas Abscesses

**DOI:** 10.7759/cureus.15908

**Published:** 2021-06-24

**Authors:** Siddharth Neelakandan, Stalin Viswanathan, Jayachandran Selvaraj, Vivekanandan Pillai, Deep Sharma, Sunitha V Chakkalakkoombil

**Affiliations:** 1 Department of General Medicine, Jawaharlal Institute of Postgraduate Medical Education and Research, Puducherry, IND; 2 Department of Orthopaedics, Jawaharlal Institute of Postgraduate Medical Education and Research, Puducherry, IND; 3 Department of Radiodiagnosis, Jawaharlal Institute of Postgraduate Medical Education and Research, Puducherry, IND

**Keywords:** emphysematous pyelonephritis, emphysematous osteomyelitis, tuberculous spondylitis, diabetes, psoas abscess

## Abstract

Emphysematous pyelonephritis (EPN) is an uncommon necrotizing infection commonly seen in people with diabetes. Emphysematous osteomyelitis (EOM) is a rare form of pyogenic osteomyelitis characterized by the presence of air in the bones. A combination of both these infections has been reported only thrice in the literature. We present the case of a middle-aged diabetic woman who had both these rare infections along with psoas abscesses, a phenomenon that has been described only once previously. The patient required prolonged hospitalization, surgical debridement and drainage, a double-J stent, and meropenem, and she subsequently achieved full recovery.

## Introduction

Emphysematous pyelonephritis (EPN) is a potentially fatal necrotizing parenchymal infection mostly seen in diabetic individuals [1]. Pyogenic spondylodiscitis is the most common spinal infection and is caused by hematogenous spread. Genitourinary causes of transient bacteremia also lead to spinal infection [2]. Emphysematous osteomyelitis (EOM) is a rare form of pyogenic osteomyelitis characterized by air in the bones [3]. As of July 2020, only 46 cases of EOM had been reported in the literature [3]. Simultaneous occurrence of EPN and osteomyelitis have been reported in only three instances previously [4-6]. We present a case involving the simultaneous presentation of EOM, EPN, and bilateral psoas abscesses in a diabetic female who was hospitalized twice and recovered uneventfully. Ours is only the second case of this nature to be reported in the literature [6]. As per clinical presentation and subtle radiological features, the condition mimicked tuberculous osteomyelitis, which was later ruled out by microbiological and histopathological evidence.

## Case presentation

A 50-year-old female from Cuddalore, Tamil Nadu, presented with complaints of anorexia, appetite loss, and back pain of two weeks' duration that had worsened over four days before admission. The pain radiated to the loin and lower limbs, and she had difficulty extending her lower limbs. She had also become drowsy two days prior to the admission. There was no history of fever, evening rise of temperature, night sweats, or any appreciable weight loss. The patient did not have a history of bowel and bladder disturbances. She had been on insulin for type 2 diabetes mellitus for the past year, with poor control and compliance. One month ago, the patient had been treated for left-sided, *Escherichia coli (E. coli)-*related EPN, which had been managed with intravenous amikacin (750 mg q24H). A double-J stent had been inserted four days after the admission for source reduction, and antibiotics had been continued for two weeks in the hospital. Due to the exigencies of coronavirus disease 2019 (COVID-19), she had been asked to complete a four-week course of antibiotics from a nearby hospital; however, the patient had discontinued treatment after a week. On examination, the patient was irritable, disoriented to time and place, was lying in bed supine with hips flexed, and was afebrile, pale, with tachycardia (heart rate of 110 beats/minute), hypotension (BP of 90/60 mmHg), and tachypnea (respiratory rate of 24 breaths/minute). Neurological examination showed hypertonia in both lower limbs with a Medical Research Council (MRC) grade of 3/5 proximally (she was able to lift her legs off the bed against gravity); extension of the knees was painful, while knee flexion, ankle dorsiflexion, and plantar flexion were MRC 4+/5. Reflexes could not be elicited due to pain, and the plantar response was equivocal. Other systemic examinations were unremarkable. The investigations are presented in Table 1. C-reactive protein analysis could not be done, as its testing was limited to patients with COVID-19.

**Table 1:** List of investigations during the hospital stay AFB: acid-fast bacilli; CBNAAT: cartridge-based nucleic acid amplification test; *E. coli: Escherichia coli*

Investigations	Day 1	Day 5	Observations	Day 21
Hemoglobin (g/L)	72	76		104
Leucocyte count (×10^9^/L)	29.03	23.25		8.56
Neutrophils (%)	90	92		74
Platelet count (×10^9^/L)	390	484		284
Blood sugar (mg/dL)	209	178		136
Urea (mg/dL)	71	59		17
Creatinine (mg/dL)	1.29	1.1		0.5
Sodium (mEq/L)	126	128		121
Potassium (mEq/L)	5.6	3.8		3.6
Calcium (mg/dL)	10.5	10.2		9.2
Alkaline phosphatase (IU/L)	720	715		228
Procalcitonin (ng/ml)		1.2		
pH	7.31			
Bicarbonate (mEQ/L)	16			
pCO_2_ (mmHg)	30			
Lactate (mmol/L)	4			
Exudate culture			*E. coli* sensitive to cefoperazone + sulbactam, amikacin, piperacillin + tazobactam, meropenem	
Blood culture (3 sets)	Sterile			
Urine culture	Sterile			
AFB staining (exudate)			Negative	
Exudate CBNAAT			TB not detected	
HbA1c (%)	14.1			
Bone biopsy			Suggestive of chronic osteomyelitis	

**Figure 1:**
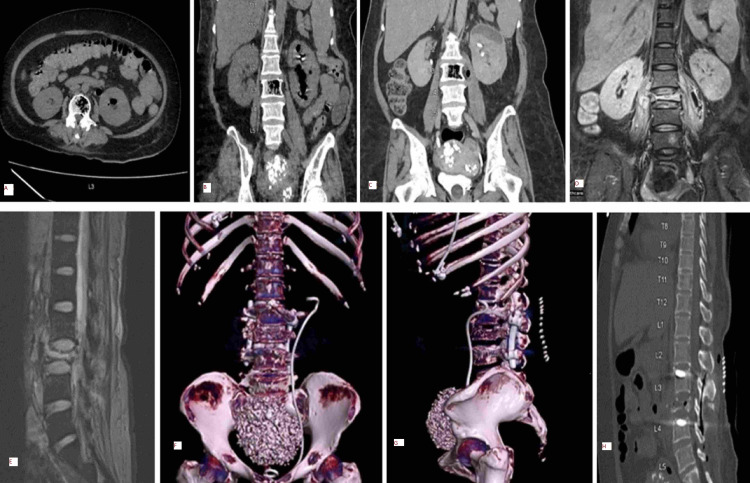
Imaging of the patient during her stay in hospital 1A: CT abdomen transverse plane shows air foci in left kidney and L3 vertebra 1B: CT abdomen coronal view shows air foci in left kidney, L3 vertebra, and enlarged left psoas muscle 1C: CT cut showing emphysematous osteomyelitis with extension to the left psoas abscess 1D: MRI during the second admission shows complete L3 collapse with psoas abscess and pyelonephritis 1E: MRI shows the abscess extending from the destroyed vertebra, extradurally, along the thecal sac with compression of cauda equina nerve roots and narrowing of neural foramina at the L2-L3 level 1F, 1G: postoperative CT volume-rendered image shows the destroyed vertebra, with the double-J stent in the left kidney 1H: sagittal CT shows post-surgical debridement status CT: computed tomography; MRI: magnetic resonance imaging

Clinically, a psoas abscess due to an inadequately treated pyelonephritis was suspected, and an ultrasonogram confirmed the presence of bilateral psoas abscesses (right: 4.2 × 1.5 cm; left: 5.9 × 2.3 cm). Cultures of blood, urine, and pus aspirated under ultrasound guidance were sent, and meropenem (1g q8H) was initiated empirically. CT kidneys performed one month prior were reviewed and showed left-sided EPN along with few air foci in L3 vertebra suggestive of EOM (Figures 1A, 1B, 1C). MRI spine performed to rule out neurological involvement on day three showed a complete collapse of L3 vertebra with preserved adjacent intervertebral discs and bilateral psoas abscesses, and the radiologist suggested the possibility of tuberculous spondylitis (Figures 1D, 1E).

However, because of previous pyelonephritis (poorly treated) and the CT findings, a diagnosis of pyogenic osteomyelitis was entertained strongly, and meropenem was continued without anti-tubercular therapy. Meanwhile, the exudate culture also grew* E. coli* sensitive to meropenem. The patient’s sensorium improved, but the back pain persisted. Due to COVID-19-related cutting down of regular OT shifts and the patient’s pre-anesthesia fitness-related issues, she underwent posterior instrumentation, decompression, and drainage of the abscess under general anesthesia only on day 11. Because of the patient's poor general condition, anterior reconstruction with posterior drainage could not be performed. Perioperatively, two RBC transfusions were given. Bone biopsy done during surgery was suggestive of chronic osteomyelitis. Five days postoperatively, the drain was removed, and she could extend both her lower limbs. Repeat CT did not show any worsening of her condition (Figures 1F, 1G, 1H). On the seventh postoperative day, she was able to sit up with support, and she started weight-bearing on the 10th postoperative day. At the end of the third week of hospitalization, she was completely asymptomatic and was walking with support. She was advised six weeks of antibiotic therapy in total, but four weeks later, she sought discharge since she had to return home due to an impending COVID-19 lockdown. After two weeks of antibiotic therapy at home, telephonically, we found that she was ambulatory and was compliant with anti-diabetic medications. At the time of the submission (eight weeks after the telephonic conversation) of this report, she had still not returned for a follow-up CT.

## Discussion

EPN was first described in the 19th century and is classified based on the findings on CT [1]. Our patient had class 3B pyelonephritis based on the involvement of the psoas muscles, which, during the first admission, was managed with medical therapy alone. Due to non-compliance, she developed symptoms of worsening psoas abscesses that limited the extension of her lower limbs. Surgical debridement and drainage of the psoas abscesses plus six weeks of antibiotics enabled her recovery. EPN is more common in women and often affects the left kidney [7]. In a series of 26 patients from India, all but two of the subjects were women, and all had diabetes mellitus [7]. Apart from diabetes, our patient did not have any poor prognostic features such as hematuria, azotemia, shock, or thrombocytopenia [1,7].

EOM is a rare variant of pyogenic osteomyelitis, which is rapidly progressive and is characterized by the presence of air in the bone; it usually involves the spine, hip, and in rare cases, peripheral bones [3]. This infection mainly spreads hematogenously from a distant focus, especially in immunocompromised states like diabetes [2]. The most common organisms isolated are Gram-negative Enterobacteriaceae, especially *E coli, Klebsiella,* and anaerobes [2,3]. The coexistence of EOM and EPN has been reported only three times in the literature [4-6]. The first reported case was that of *Klebsiella*-related EPN and EOM in a diabetic patient who died due to septic shock and multiorgan dysfunction [4]. The second report described two cases; the first was of a diabetic individual with renal abscess and EOM of L3-L5 vertebral bodies whose follow-up details are not known [5]. The second patient, also with diabetes, had bilateral EPN and EOM of the clavicle and infection of the pectoralis major and had no reported complications during the follow-up [5]. The third case report in literature was similar to ours, with the patient having *E coli*-related EPN, vertebral EOM, and bilateral psoas abscesses [6].

Tubercular spondylitis is a common extra-pulmonary manifestation prevalent in India; it is associated with significant morbidity and most commonly involves the spine in immunocompromised patients such as people living with HIV (PLHIV) and diabetes [8]. Though TB spondylitis has a chronic course, it can be clinically unnoticed and can present acutely as compressive myelopathy, radiculopathy, or as a psoas abscess [9]. Pyogenic infection usually disseminates through vascular arcades in the spine; as a result, there is early involvement of the intervertebral discs and then the adjacent vertebral body. In contrast, tuberculosis disseminates through the Batson’s paravertebral plexus, and the anterior part of the vertebral body is affected with disc sparing (which we considered in our patient during her current admission). As a result, the tubercular abscess spreads through the space between the anterior longitudinal ligament and vertebral body to the adjacent vertebral body, involving the lower part of the upper vertebra and the upper part of the lower vertebra disc, leading to complete destruction. In the later stages, pyogenic osteomyelitis can resemble TB osteomyelitis. Hence, a careful history along with a clinical and radiological examination and the support of microbiological and histopathological evidence helps in differentiating between these two clinically important infections.

## Conclusions

Coexisting EPN, EOM, and psoas abscess is exceedingly rare and necessitates multidisciplinary management involving internists, nephrologists, urologists, radiologists, orthopedic surgeons, and physiotherapists. Back pain with psoas abscess with or without neurological deficit can be present in either pyelonephritis or vertebral osteomyelitis and requires an early diagnosis to initiate aggressive treatment and prevent sequelae. Prolonged hospitalization in the time of COVID-19, where there is an acute bed shortage, must be stressed to prevent bone and kidney complications related to uncontrolled diabetes.
